# Supplementary material for: Patterns of Intron Gain and Loss in Fungi
Source: PLoS Biol. 2004 Nov 30;2(12):e422. doi: 10.1371/journal.pbio.0020422 (PMC532390; doi:10.1371/journal.pbio.0020422)
Supplement: Table S1 — Also available at http://genes.mit.edu/NielsenEtAl/. (4.3 MB ZIP). [file pbio.0020422.st001.zip › NielsenEtAl/html/1443.html]

AN6731.1.NCU05259.1.MG01790.1.FG06184.1


```
 CLUSTAL W (1.82) Multiple Sequence Alignments - Introns Inserted


Sequence 1: NCU05259.1	477 aa
Sequence 2: MG01790.1	475 aa
Sequence 3: FG06184.1	475 aa
Sequence 4: AN6731.1	455 aa
Alignment Length: 479 aa
Number Identitical Residues: 294 aa
Alignment Score (without introns) 14556


MG01790.1 	MASSSSS---VPELAAAFPDGTTDFKPMR-NTKGYDVSKPHISETPMTLKNWH~KHVNWL
NCU05259.1	MASTSATDIPAEVQAVNVPDGTTDFIPLR-GKK-YDLKKPHITELPITWGNWY1KHVNWL
FG06184.1 	MATATAAG----KNASPFPDGTKDYVPLRAGAAKKDTNKPHISDTPMTWSNWP~QHINWL
AN6731.1  	MSAPTAD--------------------IR--ARAPEAKKVHIADTAINRHNWY~KHVNWL
          	*::.::                     :*      : .* **:: .:.  **  :*:***

MG01790.1 	NTTFILFVPLAGLISTYWVPLQWKTAVWAVVYYFNTGLGIT~AG1YHRLWAHSSYKASLP
NCU05259.1	NTTFIIFVPLAGLISAYWIPAQTKTILFSIAYYYFAGLGIT1AG~YHRLWAHTSYKATLP
FG06184.1 	NTTLVVFVPLMGFISAYWVPLQLKTALWAVFYYVHTGLGIT~AG1YHRMWSHSAYKGTTP
AN6731.1  	NVFLIIGIPLYGCIQAFWVPLQLKTAIWAVIYYFFTGLGIT~AG1YHRLWAHCSYSATLP
          	*. ::: :** * *.::*:* * ** :::: **  :***** ** ***:*:* :*..: *

MG01790.1 	LKIYLAAVGAGAVEGSIRWWSNGHRAHHRYTDTEKDPYSVRKGLLYSHMGWMLLKQNPKK
NCU05259.1	LKIWLAAGGAAAVEGSARWWSRDHRAHHRYTDTDKDPYSVRKGLLYSHIGWMVMKQNPKR
FG06184.1 	LKIYLAAVGAGAVQGSIRWWSYGHRVHHRYTDTDKDPYSVRKGLMYSHMGWMVLKQNPKR
AN6731.1  	LRIWLAAVGGGAVEGSIRWWARDHRAHHRYTDTDKDPYSVRKGLLYSHLGWMVMKQNPKR
          	*:*:*** *..**:** ***: .**.*******:**********:***:***::*****:

MG01790.1 	QGRTDITDLNEDPVVVWQHRNFLKCVIFMALVFPTLVAGLGWGDYWGGFIYGGILRVFFV
NCU05259.1	IGRTDITDLNEDPVVVWQHKHYIKCVIVMALIVPTLICGLGWGDYAGGFIYGGILRIFFI
FG06184.1 	QGRTDITDLNEDAVVVWQHKNYIKCVLFMALAFPAIVAGLGWGDWWGGLVYAGILRVCFV
AN6731.1  	IGRTDISDLNEDPVVVWQHRNYLKVVFTMGLAVPMLVAGLGWGDWLGGFVYAGILRIFFV
          	 *****:*****.******::::* *: *.* .* ::.******: **::*.****: *:

MG01790.1 	QQATFCVNSLAHWLGDQPFDDRNSPRDHVITALVTLGEGYHNFHHEFPSDYRNAIEWYQY
NCU05259.1	QQATFCVNSLAHWLGDQPFDDRNSPRDHVITALVTLGEGYHNFHHEFPSDFRNAIEWWQY
FG06184.1 	QQATFCVNSLAHWLGDQPFDDRNSPRDHVITALVTLGEGYHNFHHEFPSDYRNAIEWWQY
AN6731.1  	QQATFCVNSLAHWLGDQPFDDRNSPRDHVITALVTLGEGYHNFHHEFPSDYRNAIEWHQY
          	**************************************************:****** **

MG01790.1 	DPTKWSIWIWKQLGLAHNLKQFRQNEIEKGRVQQLQKKLDQKRAKLDWGIPLEQLPVVSW
NCU05259.1	DPTKWCIWVWKQLGLAYDLKKFPHNEIEKGRLQQQQKKLDQKRATLDWGIPLENLPVVSW
FG06184.1 	DPTKWSIWLWKQLGLAYELKEFRANEIEKGRVQQLQKKLDQKRATLDWGIPLEQLPVVDW
AN6731.1  	DPTKWSIWAWKQLGLAYDLKKFRANEIEKGRVQQLQKKLDRKRATLDWGTPLDQLPVMEW
          	*****.** *******::**:*  *******:** *****:***.**** **::***:.*

MG01790.1 	DDFVEQSKNGKAWIAVAGVIHDVGDFIKDHPGGRALINSAIGKDATAIFNGGVYNHSNAA
NCU05259.1	DDFVQESKNGKAWIAVAGVIHDVGKFIQDHPGGKALINSAIGKDATAIFNGGVYNHSNAA
FG06184.1 	DDFVAQSKAGKGLVAIAGVIHDVTDFIKDHPGGKALINSAIGKDATAIFNGGVYLHSNAA
AN6731.1  	DDYVEQAKNGRGLVAIAGVVHDVTDFIKDHPGGKAMISSGIGKDATAMFNGGVYYHSNAA
          	**:* ::* *:. :*:***:*** .**:*****:*:*.*.*******:****** *****

MG01790.1 	HNLLSTMRVGVLRGGCEVEIWKRAQSENKDVSTVVDSSGNRIVRAGGQATKVVQPVPGAQ
NCU05259.1	HNLLSTMRVGVIRGGCEVEIWKRAQAENKDIATVTDSSGKKIVRAGLQPTRVTPPVTTAD
FG06184.1 	HNLLSTMRVGVLRGGCEVEIWKRAQFENKDITYMNDSAGQRIIRAGSQATKIVQPAASAD
AN6731.1  	HNLLSTMRVGVIRGGCEVEIWKRAQKEN--VEYVRDGSGQRVIRAGEQPTKIPEPIPTAD
          	***********:************* **  :  : *.:*::::*** *.*::  * . *:

MG01790.1 	AA
NCU05259.1	AA
FG06184.1 	AA
AN6731.1  	AA
          	**
```
